# A Longitudinal Study of Adolescent Mental Health and Quality of Life as Predictors of College Physical Health, Mental Health, and Gluten-Free Diet Adherence in Celiac Disease

**DOI:** 10.3390/nu17223568

**Published:** 2025-11-14

**Authors:** Tierra L. Mosher, Lilly Jill Su, Javier A. López-Rivera, Ritu Verma, Kate Keenan, Hilary Jericho

**Affiliations:** 1 Department of Pediatrics, Stanford University, Palo Alto, CA 94304, USA; lillysu@stanford.edu; 2 Division of Gastroenterology, Hepatology, and Nutrition, Stanford University, 750 Welch Road, Suite 116, Palo Alto, CA 94304, USA; javilop@stanford.edu; 3 Department of Pediatrics, Division of Gastroenterology, Hepatology, and Nutrition, Celiac Disease Center, University of Chicago Medicine, Comer Children’s Hospital, 5721 S. Maryland Avenue, Chicago, IL 60637, USA; fverma@bsd.uchicago.edu (R.V.); kkeenan@bsd.uchicago.edu (K.K.)

**Keywords:** celiac disease (CeD), adolescents, mental health, quality of life, college, young adults, adherence rates

## Abstract

**Background/Objectives:** To determine whether adolescent (T1) mental health, quality of life, and adjustment to celiac disease (CeD) are associated with college-age (T2) perceived physical and mental health and gluten-free diet (GFD) adherence. **Methods:** In 2015, adolescents with CeD (*n* = 101, T1) completed standardized surveys assessing mental health (CSI-4), quality of life (PedsQL), and adjustment to CeD (CDDUX). Five years later, participants ≥18 years self-reported GFD adherence and physical and mental health perception in college (*n* = 59, T2). Patients who were current or recent college students that provided complete data at both time points were analyzed (*n* = 43) using Kendall’s tau to test: concurrent associations among T2 perceived physical/mental health and GFD adherence; and prospective associations between T1 measures and T2 perceived outcomes. **Results:** Higher T1 CSI-4 and PedsQL scores were negatively correlated with T2 perceived physical health (*τ* = −0.31, *p* = 0.02 and *τ* = −0.28, *p* = 0.04, respectively). There was trending association between T1 PedsQL and T2 mental health perception (*τ* = −0.23, *p* = 0.06). T2 physical and mental health perception were positively correlated (*τ* = 0.41, *p* = 0.001). No significant associations emerged between T1 measures and T2 GFD adherence, nor between T2 health perception and GFD adherence, although T2 physical health perception positively trended with GFD adherence (*p* = 0.78). **Conclusions:** Adolescents with CeD reporting more depressive symptoms or lower quality of life feel less physically and mentally healthy when in college. In college, feeling physically healthy aligns with feeling mentally healthy, although neither clearly predicts GFD adherence. Early mental health screening in adolescents with CeD may support transitions to independent dietary management.

## 1. Introduction

Mental health continues to be a prominent area of focus in pediatric research given the sharp increase in rates of depression, anxiety, and suicidality among youth [1]. Adolescent patients in particular currently need more attention than ever. According to the US Agency for Healthcare Research and Quality, children aged 10 to 19 years represent 13% of the global population living with a diagnosable mental health condition [1]. Moreover, the burden is not evenly distributed. Adolescent patients who must also manage a chronic medical disorder face the dual challenge of juggling their illness with an already stressful time period in life, making them an especially high-risk population [2]. The transition from adolescence into early adulthood magnifies this pressure and is a critical time point both for illness management and mental health [2,3]. Prior research confirms that adolescent and college patients with CeD have higher rates of depression and report lower quality of life, but there has been minimal investigation into the same cohort from adolescence into young adulthood despite this developmental interval being one in which responsibility for strict diet management transfers abruptly from parent to patient thereby increasing risk of adherence issues [4]. CeD is unique in that treatment is nutritional, with lifelong adherence to a strict gluten-free diet being essential to preventing villous atrophy [5]. Lack of adherence can lead to symptoms of recurrent abdominal pain, diarrhea, and weight loss, and long-term risks such as malnutrition, micronutrient deficiencies, and osteoporosis [5]. These historical findings in the literature related to psychological health decline within this patient population have implications for GFD adherence warranting further investigation. By following a well-characterized sample for five years, our study uniquely captures whether psychosocial signals recorded in high school forecast health perception and dietary adherence once students are more autonomous in the college setting. We aim to determine whether adolescent (T1) measures of depressive symptoms and quality of life are associated with T2 physical and mental health perception and GFD adherence; and whether, among college students with CeD (T2), self-reported physical and mental health are concurrently associated with GFD adherence. Addressing these questions may pinpoint modifiable psychosocial targets to reinforce diet adherence and overall well-being during a vulnerable transition point in a patient’s life.

## 2. Materials and Methods

### 2.1. Participants and Study Design

Adolescents diagnosed with CeD were originally recruited in 2015 from the University of Chicago Pediatric Gastroenterology clinic. Eligible participants were required to meet the European Society for Pediatric Gastroenterology, Hepatology, and Nutrition (ESPGHAN) diagnostic criteria (T1) [6]. Participants provided informed consent for both initial participation and future contact which was performed by electronic mail or telephone. Five years later at T2 (2020), all original participants aged ≥18 years and currently attending or having recently graduated from college were invited to complete a follow-up survey assessing GFD adherence and self-reported physical and mental health perception. Of the 101 initial enrollees, 59 participants completed the T2 survey, 47 participants met the college criterion, and 43 provided complete data at both time points. All research activities including encrypted data storage were conducted at the University of Chicago, and de-identified datasets were subsequently transferred to Stanford University under a formal Data Use Agreement in collaboration with the University of Chicago.

### 2.2. Measures

For T1 symptom investigation, the instruments used to evaluate depressive symptoms included Child Symptom Inventory-4 Major Depressive Disorder subscale (CSI-4), which captures mood, appetite, and sleep disturbances, with a score range between 0 and 25, and higher scores indicating greater symptom severity [7]. In this same population, quality of life was measured utilizing the Celiac Disease Quality of Life (CDDUX) survey to assess disease-specific concerns, with a score range between 0 and 100, where higher scores indicate higher disease-specific quality of life [8]. Additionally, the Pediatric Quality of Life Survey (PedsQL) survey was used to assess physical, emotional, social, and school functioning, with a score range from 0 to 100, and higher scores indicating higher quality of life [9]. For T2 (college) symptom investigation, a study-specific nine-item Likert survey captured three domains: perceived physical health, perceived mental health, and GFD adherence. Response categories ranged from 1 = ‘very poor/never’ through 5 = ‘excellent/always’. Responses within each domain were summed to create aggregate scores wherein higher scores mean better perceived health or stronger self-reported GFD adherence. All T1 and T2 raw scores were converted to their published metric or summed Likert totals.

### 2.3. Statistical Analysis

Kendall’s tau correlations were computed to test bivariate associations between each T1 measure (CSI-4, PedsQL, CDDUX) and every T2 outcome (perceived physical health, perceived mental health, GFD adherence). The same statistic was used for within-time point associations among the three T2 domains. Analyses were carried out in R v. 4.3.3. Statistical significance was defined a priori as α = 0.05. To control the false-discovery rate arising from multiple correlation tests, raw *p*-values were adjusted with the Benjamini–Hochberg procedure. No additional modeling or sensitivity analyses were performed. Descriptive data are reported as mean ± standard deviation (SD).

## 3. Results

**Figure 1:**
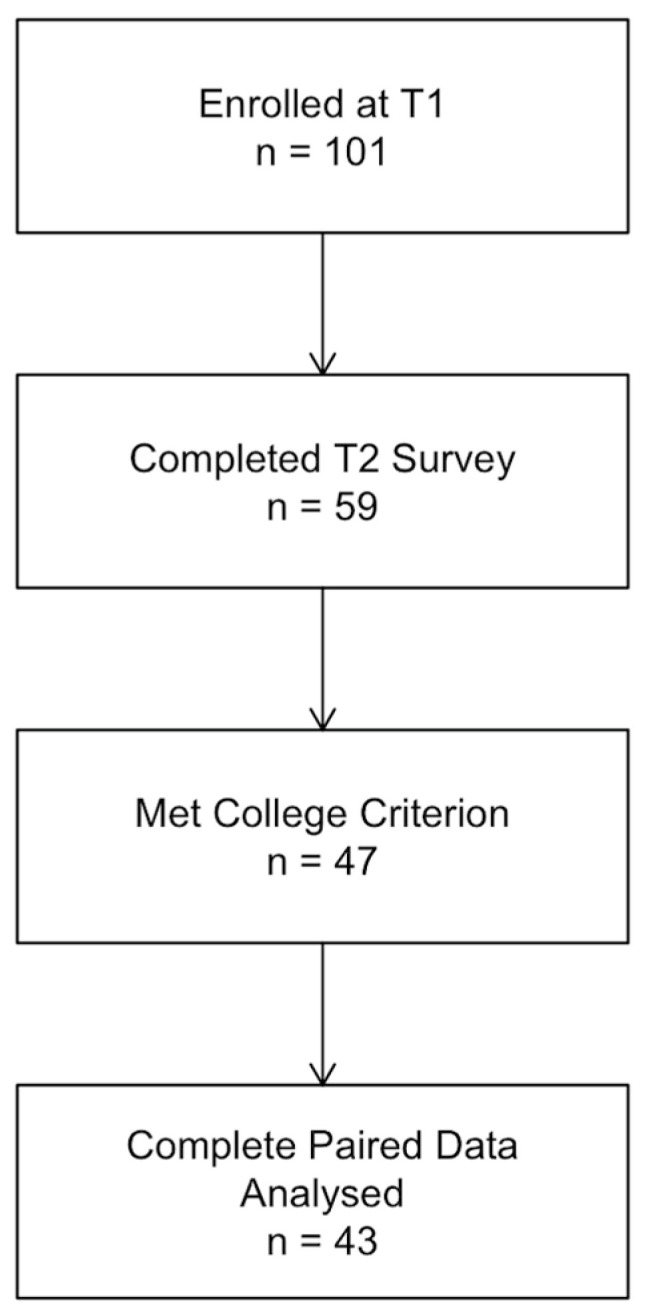
CONSORT diagram. CONSORT-style flow diagram of participant recruitment and retention.

Figure 1 CONSORT diagram details the participant flow. Of the 101 original T1 participants who completed baseline surveys in 2015, 59 (58%) responded to the 2020 follow-up invitation at T2. Among these respondents, 47 confirmed that they were either currently in or recently graduated from college, thereby satisfying our T2 inclusion criterion. Finally, 43 provided complete, analyzable datasets for both time points. The final analytic cohort (*n* = 43) therefore represents 42% of the original sample and was composed of 13 (30.2%**)** males.

### 3.1. Associations at T2

Kendall’s tau showed no significant association between T2 physical health and GFD adherence (*τ* = 0.032, FDR-adjusted *p* = 0.78), or between T2 mental health perception and GFD adherence (*τ* = −0.077, FDR-adjusted *p* = 0.77). There was significant association between T2 physical health and mental health (*τ* = 0.41, FDR-adjusted *p* = 0.001).

### 3.2. Prospective T1 to T2 Associations

**Figure 2:**
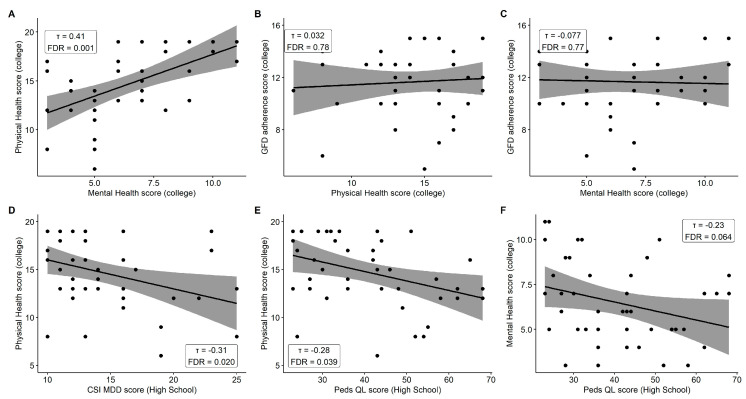
T1 and T2 Associations and Trends. (**A**) Physical and mental health perception in college. (**B**) College physical health perception and GFD adherence. (**C**) College mental health perception and GFD adherence. (**D**) High school mental health and college physical health perception. (**E**) High school quality of life and college physical health perception. (**F**) High school quality of life and college mental health perception.

Higher T1 CSI-4 scores were moderately negatively correlated with lower T2 physical health perception (*τ* = −0.31, FDR-adjusted *p* = 0.02). Higher T1 PedsQL scores showed a similar moderate negative correlation with T2 physical health perception (*τ* = −0.28, FDR-adjusted *p* = 0.04). There was a suggestive positive trend between T1 PedsQL and T2 mental health perception (*τ* = −0.23, FDR-adjusted *p* = 0.06). A non-significant positive trend was also found between T2 physical health perception and T2 self-reported GFD adherence (*τ* = 0.032, FDR-adjusted *p* = 0.78). All *p* values reflect Benjamini–Hochberg adjustment. Complete correlation coefficients and means (SD) for each measure appear in Figure 2 and Table 1.

## 4. Discussion

For patients with celiac disease (CeD), strict lifelong adherence to a gluten-free diet (GFD) is not only the cornerstone of treatment, but more importantly it is non-negotiable. Not adhering to this diet impacts patients through uncomfortable daily symptoms and long-term increased risk of poor nutrition status, iron deficiency anemia, and suboptimal bone health [5]. Literature consistently supports that the time period from late adolescence into early adulthood is one in which patients are at increased risk for poor adherence to treatment of chronic illnesses and development of mental health concerns [2,3]. There is an importance in highlighting any associations that may exist between mental health and GFD adherence that can be targeted to better support our patients in their transition to young adulthood. Although there are many factors that can contribute to poor adherence, limited data exists on the relationship between mental health perception and quality of life in adolescence (T1) and its subsequent impact during college (T2). This is the aim of our study, and this analysis is among the first to investigate this question. This longitudinal cohort demonstrates that adolescent psychosocial status, most notably depressive symptoms and disease-specific quality of life, carries forward to college, which ultimately could shape how emerging adults perceive their physical well-being.

In our analyses, we observed that there was no statistically significant association detected between physical and mental health perceptions in T2 and GFD adherence. Nevertheless, the directionality of the coefficients suggested a modest positive trend in which better physical health perception may be associated with dietary compliance. In contrast, psychosocial status recorded in the initial time period for data collection was more telling: higher T1 depressive symptoms and lower quality of life scores were associated with lower physical health in college.

There is literature supporting the correlation between mental health and its impact on management of chronic illness [10,11]. While our data suggests that positive T2 physical and mental health perceptions are correlated with one another, neither appears to have a positive correlation to T2 GFD adherence. Several pathways could mediate this effect in CeD. We suspect these findings are secondary to one of three possible reasons: (1) there truly is no relationship between GFD adherence and mental health; (2) adaptation in college including social pressures, dining logistics, and stress which were not controlled for in this study are impacting the findings; (3) the individual’s report of their GFD adherence is not truly aligned with their actual dietary adherence; or (4) our findings do not accurately depict the relationship between CeD dietary adherence and mental health as a result of small sample size in the setting of difficulty retaining participant volume in the follow up study. Future evaluation of this comparison should involve a larger sample size to either support or disprove our current findings. Additionally, follow up studies could include evaluating tissue transglutaminase immunoglobulin A (TTG IgA) levels in conjunction with perceptions of physical and mental health survey data for more objective findings [12].

Unexpectedly, adolescents (T1) who reported the best quality of life in high school later rated their physical health lower in college. Several mechanisms may underlie this inverse T1–T2 association. First, adolescents with exceptionally high baseline PedsQL scores may encounter an ‘expectation–reality’ gap once parental scaffolding disappears and they must self-manage a strict GFD amid new academic and social demands [13]. Secondly, emerging adults often face heightened stress from competitive coursework and unfamiliar peer networks. These are just some of the conditions have been that have been shown to erode perceived mental health even among previously high well-being students [14]. Finally, campus dining environments vary widely in gluten-free labeling and cross-contact controls, which poses practical barriers that can quickly undermine earlier optimism. Together, these contextual stressors offer a possible explanation for why students who initially felt healthiest became comparatively vulnerable during the college transition.

One limitation of our study is the lack of assessment for which T1 participants with negative mental health scoring received mental health interventions (therapy or medication). Additionally, there were difficulties with patient retention resulting in a small sample size notable by only 59 of the 101 original adolescents who completed follow-up which we feel could be impacting our study results. The lack of participant retention from adolescence into college was likely largely secondary to outdated contact information and the logistical constraints of the early COVID-19 pandemic. Despite our efforts, a 42% attrition rate may introduce selection bias. Strengths of our study include the use of validated psychosocial questionnaires in T1, the five-year follow up window that highlights the critical transition period from high school to college, and the rigorous statistical analyses used to assess our data and support our findings. A goal for future studies includes repeating the aforementioned screening tools in adolescent patients, employing the use of additional resources and therapies, and then prospectively following these patients through college to better understand the impact of these tools on patients physical and mental health in these critical points of life transition.

In summary, our results highlight an urgent need to probe more deeply into the intricate, bidirectional relationship between mental and physical health in adolescents with celiac disease. The data presented here serves as a call to action for routine mental health screening and timely intervention within pediatric CeD care pathways to both ease emotional distress and support patients in their dietary adherence during a high-risk transition period in their lives. Additionally, the study reinforces that management of chronic, nutrition-dependent conditions in young adulthood must move beyond an isolated approach at dietary guidance and instead toward an integrated model in which emotional resilience, psychosocial support, and perception of mental and physical health are addressed. Although we have not differentiated a causal relationship between mental health and dietary adherence, the associations we document support the need for further investigation. By highlighting this topic, we hope to catalyze future longitudinal and interventional studies that can refine the transition process from adolescence into young adulthood, and inform guideline development, thereby enhancing both the mental and physical well-being of adolescents and young adults living with CeD.

## Figures and Tables

**Table 1:** T1 and T2 Associations.

Time Period	Score	Mean (SD)	Correlation with T2 Scores		
			Physical Health	Mental Health	GFD Adherence
**T1**	CDDUX	31.05 (±4.48)	*τ* = 0.17, FDR = 0.13	*τ* = 0.19, FDR = 0.13	*τ* = 0.22, FDR = 0.13
	CSI-4	14.28 (±4.31)	*τ* = −0.31, FDR = 0.02	*τ* = −0.18, FDR = 0.20	*τ* = −0.13, FDR = 0.28
	PedsQL	40.81 (±13.38)	*τ* = −0.28, FDR = 0.04	*τ* = −0.23, FDR = 0.06	*τ* = 0.03, FDR = 0.78
**T2**	Physical Health Perception	14.72 (±3.57)	--	*τ* = 0.41, FDR = 0.001	*τ* = 0.03, FDR = 0.78
	Mental Health Perception	6.49 (±2.26)	*τ* = 0.41, FDR = 0.001	--	*τ* = −0.08, FDR = 0.77
	GFD Adherence	11.69 (±2.52)	*τ* = 0.03, FDR = 0.78	*τ* = −0.08, FDR = 0.77	--

## Data Availability

The raw data supporting the findings of this study will be made available by the corresponding authors only upon request due to ethical reasons.

## Abbreviations

The following abbreviations are used in this manuscript:

CeD	Celiac Disease
GFD	Gluten-free Diet
CSI-4	Child Symptom Inventory-4 Major Depressive Disorder subscale
CDDUX	Celiac Disease DUX questionnaire
PedsQL	Pediatric Quality of Life Inventory
TTG IgA	Tissue Transglutaminase Immunoglobulin A
